# A Historical Examination of Military Records of US Army Suicide, 1819 to 2017

**DOI:** 10.1001/jamanetworkopen.2019.17448

**Published:** 2019-12-13

**Authors:** Jeffrey Allen Smith, Michael Doidge, Ryan Hanoa, B. Christopher Frueh

**Affiliations:** 1 Department of History, University of Hawaii, Hilo; 2 The Department of Defense, Arlington, Virginia; 3 University of Hawaii, Hilo; 4 Department of Psychology, University of Hawaii, Hilo; 5 Department of Psychiatry, University of Texas Health Sciences Center, Houston

## Abstract

**Importance:**

Suicide rates among active-duty personnel in the US military have increased substantially since 2004, and numerous studies have attempted to contextualize and better understand this phenomenon. Placing contemporary examinations of suicides among active-duty personnel in the US Army in historical context provides opportunities for joint historical and epidemiological research to inform health care professionals and policy makers.

**Objectives:**

To consolidate data on suicide rates among active-duty personnel in the US Army as far back as historical records allow and to identify historical trends to separate them from more acute causal factors.

**Design, Setting, and Participants:**

This cross-sectional study included all active-duty service members in the US Army from 1819 to 2017 as identified and detailed in US government publications, studies, and journal articles. Empirical data were extracted from US government publications and journal articles published from 1819 to 2017. Data collection and analysis were completed between July and August of 2019.

**Exposure:**

Suicide.

**Main Outcomes and Measures:**

Suicide rates per 100 000 individuals.

**Results:**

Starting in 1843, the overall trend in annual suicide rates among active-duty service members in the US Army increased, with a peak rate of 118.3 per 100 000 in 1883. From that historical high point, the rate decreased in 3 successive waves, each corresponding to the end of the following wars: the Spanish-American War (1898), World War I (1914-1918), and World War II (1939-1945). The latter had the historically lowest rate of 5 per 100 000 in 1944 to 1945. During the Cold War (approximately 1945-1991), the rate generally stabilized in the low teens to midteens (ie, 10-15 per 100 000). The rate increased again during the Afghanistan and Iraq Wars, increasing to 29.7 per 100 000 in 2012. From 2008 to present, the annual rate has remained within the range of 20.2 to 29.7 per 100 000.

**Conclusions and Relevance:**

This study represents the most extensive historical examination of suicides in the US Army to date. By taking a long-term historical approach to suicide among active-duty personnel in the US Army, this study affords future researchers a new analytical tool and an additional perspective from which to better differentiate long-term and historical trends from more short-term and temporary causal factors.

## Key Points

**Question:**

How do suicide rates among active-duty US Army service members in the 21st century compare with those in the 20th and 19th centuries?

**Findings:**

This cross-sectional study, which includes data on all active-duty personnel in the US Army from 1819 to 2017, documented trends in suicide rates. The findings suggest that suicides historically decreased during wartime, but that pattern seems to have changed during the wars in Vietnam, Iraq, and Afghanistan.

**Meaning:**

The results of this study demonstrate the usefulness of increased historical-epidemiological partnerships to better separate long-term causes from more short-term factors and to aid in understanding the current spike in suicides among active-duty personnel in the US Army.

## Teaser

This cross-sectional study consolidates data on suicides among active-duty personnel in the US Army from 1819 to 2017 and identifies historical trends to separate them from more acute causal factors.

## Introduction

Since 2004, the suicide rate among active-duty personnel in the US military has risen substantially.^1^ Because of the recent nature of this phenomenon, current studies generally use relatively little historical perspective or data. Placing today’s medical and military explanations and theories in historical context is fundamental to gaining a deeper understanding of the current phenomenon of increases in suicides among active-duty military personnel. By incorporating historical context and data to a greater degree, researchers, medical professionals, and the US military could draw potentially new inferences that further contextualize contemporary data. Additionally, the analysis of historical records affords the US military opportunities to test current theories and strategies against the historical record of its largest branch during 3 centuries (ie, the 19th, 20th, and 21st) and through a number of significant military engagements and wars. Modern models and explanations of suicide among active-duty personnel in the US Army that account for the past may gain greater accuracy in understanding the present and preparing policy makers, officials, and clinicians for the future.

In a 2012 study using historical medical records,^2^ we found that data gathered by the US military during the US Civil War provided a conservative estimated suicide rate of between 8.74 and 14.54 per 100 000 among white individuals who belonged to the active-duty Union Army. This rate is considerably lower than the estimated suicide rates among active-duty personnel of the US military since 2004. Available US Army historic medical records provide data sources for longer periods of study, which affords opportunities to identify long-term and historical trends and separate them from more short-term and temporary causal factors. Long-range data trends suggest that the US Army is not insulated from social, cultural, and economic shifts, as previously held notions presumed. Furthermore, a historical approach to suicidality reveals potentially fruitful avenues for joint historical-epidemiological research that could inform health care professionals and policy makers.

## Methods

### Study Overview

Historical cross-sectional data were extracted from US government publications and journal articles published from 1840 to 2018. Specifically, 19th-century data came from US Army Surgeon General annual and periodic statistical reports,^3,4,5^ and the *Medical and Surgical History of the War of Rebellion, 1861-1865*.^6^ Data from the 20th and 21st centuries incorporated sources from US military health and personnel readiness reports and academic journals. Overall, raw annual rates for the 19th century until World War I came from US Army Surgeon General publications, while the rates for World War I until 2017 draw on additional military medical sources. These latter sources were compiled by 1 of us (M.D.), a Department of Defense historian who worked alongside Department of Defense epidemiologists in compiling data beginning in 2001. The author (M.D.) had direct access to the Department of Defense Suicide Event Report data as well as to the epidemiologists who created it. In historically contextualizing the US Army’s data, the author (M.D.) tracked the increase in sophistication over time. Because the data in this study were publicly available, deidentified historical data, this research was exempt from institutional review board review per Common Rule, and approval of ethical considerations for the protection of human subjects was not required nor were informed consent forms collected. This study complies with Strengthening the Reporting of Observational Studies in Epidemiology (STROBE) reporting guideline.

### Data Sources

While efforts to create a singular repository for tracking military suicide are relatively new, individual branches of the US military have tracked suicide among active-duty personnel with varying degrees of interest and analysis since the early 19th century. Although undertaken in an effort to identify causal links between health and climate to formulate “a system of medical geography,” the *Statistical Report on the Sickness and Mortality in the Army of the United States* used US Army “vital statistics of the troops extending over a period of twenty years”^3^ (ie, 1819-1839). In doing so, the statistical report explicitly recorded the first US Army suicides in the decade from 1829 to 1839. Unfortunately, the Surgeon General Office pooled the suicide data into multiyear date ranges associated with specific forts and barracks across the United States because the military was more concerned with associated data and geography than with investigating armywide trends.

Nevertheless, seeing the value in producing these reports more frequently, the next *Statistical Report on the Sickness and Mortality in the Army of the United States*^4^ covered 16 years (1839-1855), and shortly thereafter, a third *Statistical Report on the Sickness and Mortality in the Army of the United States*^5^ examined 5 years (1855-1860). By 1843, the US Army began tracking suicide among service members with enough confidence to include annual totals in their published statistical reports. In 1856, the introduction to the second *Statistical Report on the Sickness and Mortality in the Army of the United States* noted that “although it was possible to exhibit with accuracy the entire amount of sickness and mortality… the utility of such statistics would not be commiserate with the very great amount of labor necessary to their competition.”^4^ In this way, the US Army Surgeon General Office acknowledged its belief in the veracity of their medical reporting, but given the time and resources needed, they questioned the utility and practicality—something US Army Surgeon General Offices in the 20th century would do for similar reasons. The office also acknowledged that the US Army medical reporting system was incapable of detailed medical tracking during the active combat of the Mexican-American War (1846-1848). This underscores the impressive improvements and expansion of capacity required to produce the medically and militarily groundbreaking *Medical and Surgical History of the War of Rebellion, 1861-1865*,^6^ a detailed record of illnesses, combat injuries, and behavioral health problems compiled by the Union Army during the significantly larger and longer US Civil War (1861-1865).

In addition to these multiyear, 19th-century statistical reports, empirical data were extracted from US Army Surgeon General Office annual reports for the remainder of the 19th century,^7,8,9,10,11,12,13,14,15,16,17,18,19,20,21,22,23,24,25,26,27,28,29,30,31,32,33,34,35,36,37^ through World War I (1914-1918), World War II (1939-1945), and the Korean War (1950-1953) until 1957 (eAppendix 1 in the Supplement).^38,39^ The US Army did not systemically concern itself with suicide rates during the 1950s and early 1960s; the publication of suicide data in Surgeon General Office annual reports became sparse following the reorganization of the US Army beginning in 1962, which significantly reduced the Adjutant General’s authority, including their publication and records management functions. For the 1960s and 1970s, this study study primarily relied on the work of US Army health analysts, clinical researchers, and epidemiologists, whose peer-reviewed research made Surgeon General Office data public.^40,41^ By the 1980s, Army epidemiologists regularly published research and data on suicides among personnel in the US Army.^42,43,44,45^ Additionally, this study used records from the Adjutant General (1990-2007), Army Suicide Event Report (2003-2007), and the Department of Defense Suicide Event Report (2008 to the present) (eAppendix 2 in the Supplement).^46,47,48,49,50,51,52^

### Data Variability and Integrity

The current study only included data that was explicitly reported as suicide in the data sources. For example, while the oldest source, *The Statistical Report on the Sickness and Mortality in the Army of the United States*,^3^ reported deaths categorized as “mania a potu,” drowning, “broken spirit,” “sudden,” “ebriety,” and “worn out by obscure chronic affections,” only deaths categorized as “suicide” were counted. We believed that because reclassification of historical reports of causes of death would have proved methodologically problematic. However, as reporting sources changed over time, so too did nomenclature; thus, euphemistic terms, such as *self-inflicted*, or Latin terms, such as *suicidium*, were also included. Concerns over the integrity of suicide data from the US Civil War era—understandable given the data’s age—have been previously addressed,^53^ including issues of the homicide to suicide ratio, contemporaneous newspaper accounts, seasonal variations, and nongovernmental data sourcing.

From 1912 through the 1970s, the US Army frequently expressed rounded numbers for suicide data in official reports and academic findings, which probably resulted from the medical service’s need to give accurate estimates of noncombat casualties. The absence of reporting data was never interpreted as an absence of suicide. Conversely, when suicide deaths and rates were folded into larger statistical categories, often including homicides and what the reports referred to as accidents, these figures were not used because there was no way to differentiate between causes of death.

Because this study views US Army suicide as a medical issue and aims to support data integrity and continuity over the centuries, US Army Surgeon General Office data were given preeminence over other data sourcing, including additional government publications. The US Army was selected for examination because it is historically the largest and, with the US Navy, the oldest of the 5 branches of the US military, with the most complete and publicly accessible data. The US Army also comprises primarily physically fit, drug-free, and healthy young and middle-aged individuals. All sources addressed suicide rates among active-duty personnel in the US Army.

### Statistical Analysis

Extracted data were recorded as annual US Army suicide rates per 100 000 active-duty personnel and graphed chronologically in Excel (Microsoft Corp). Because of the smaller size of the US Army in the 19th century, the military often reported suicide rates per 10 000 individuals. None of the data sources reported statistical significance or confidence internals. No additional statistical testing was conducted. While the military sometimes expressed data or annual rates in the 19th century to the second decimal place (perhaps to signal its confidence in the returns), 20th-century data were more often reported as whole numbers. By the 21st century, US Army reporting generally reported data to the first decimal point.

## Results

The *Statistical Report on the Sickness and Mortality in the Army of the United States*^3^ represented the first published US Army statistical medical report to acknowledge suicide among active-duty personnel in any meaningful way. Although the medical records consulted for the publication from 1819 to 1828 failed to explicitly mention suicide, the medical returns from 1829 to 1839 attributed 11 deaths to suicide. However, as the statistical report’s main goal was “the development of laws of climate, and the application of these laws to the elucidation of disease,”^3^ the report failed to state annual returns. Instead, it focused on a geographically driven categorization of the location of specific US Army forts or barracks and reported their respective suicide returns as multiyear totals encompassing the entire tenure of occupation. Therefore, the first annual, statistically usable suicide rate for the US Army was that of 1843, when 1 suicide from a US Army population of nearly 10 000 resulted in a rate of 10.38 per 100 000 service members (Figure).

**Figure.**
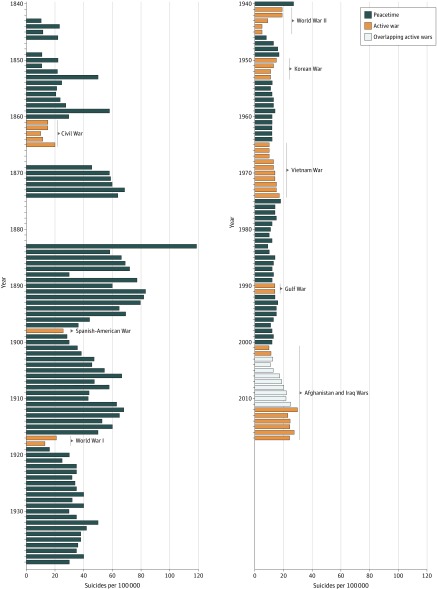
Suicides Rates Among Active-Duty Personnel in the US Army, 1843-2017

After 1843, the overall trend in the annual suicide rate among active-duty personnel of the US Army increased. In 2 instances (ie, 1866-1868 and 1875-1882), we were unable to differentiate suicide from other causes of death; therefore, we were unable to determine the suicide rates for these years. The suicide rate was highest in 1883, at 118.29 per 100 000 from a force of nearly 23 000 service members (Figure). From the historical high point of 1883, the rate decreased in 3 successive waves, corresponding to the end of the following wars: the Spanish-American War (1898), World War I (1914-1918), and World War II (1939-1945). The historically lowest rate of 5 per 100 000 service members was recorded in 1944 and 1945 (Figure). During the Cold War (approximately 1945-1991), the rate stabilized in the low teens to midteens (ie, 10-15 per 100 000 service members), with the highest rate in 1975, with 18 per 100 000 when the Vietnam War (1955-1975) was ending (Figure). It was not until the early 21st century that the rate began to increase again during the Afghanistan and Iraq Wars, with the highest rate in 2012 at 29.7 per 100 000 service members (Figure). From 2008 to the present, the annual rate has remained between 20.2 and 29.7 per 100 000 service members (Figure).

Suicides among active-duty personnel in the US Army continued to decrease throughout the 20th century, decreasing substantially during World War II. From 1938, with a rate of 40 per 100 000 service members, suicide rates among active-duty personnel in the US Army decreased to a low of 5 per 100 000 service members in 1945 (Figure). The suicide rates entered a new paradigm from 1946 to 2005. During this time, Army suicide rates remained between 10 and 15 per 100 000 service members and spiked to 18 per 100 000 service members in 1975 (Figure). Beginning in 2004, suicides increased substantially and, in 2008, exceeded 20 per 100 000 service members for the first time since 1940. Rates continued to increase until 2017 (Figure).

## Discussion

To our knowledge, this historical cross-sectional study represents the most extensive historical examination of suicide among active-duty personnel in the US Army to date and, by extension, is among the largest studies of military suicide. By taking a long-term (ie, nearly 200 years) historical approach to suicide among service members in the US Army and analyzing data over longer periods, this study provides future researchers a new analytical tool to aid in better differentiating long-term and historical trends from more short-term and temporary causal factors.

When examining the data from a historical vantage point, there appears to be a general decrease in suicide rates among active-duty personnel during active combat until the wars in Vietnam, Iraq, and Afghanistan. The US Civil War, the Spanish-American War, World War I, World War II, and the Korean War were associated with decreased suicide rates among active-duty personnel in the US Army (Figure; eAppendix 3 in the Supplement). However, a noticeable change in this trend began with the Vietnam War and continued through the 21st-century wars in Iraq and Afghanistan (Figure).

Recent suicide patterns seem to indicate the development of a new paradigm, as the Department of Defense Suicide Event Report details only approximately half of those who die by suicide.^52^ As historical trends appear to show decreases in wartime suicide rates and as suicide is multifactorial, the findings of this study suggest that factors away from the battlefield may be associated with the change in suicide rates during active combat and among personnel in the US Army. However, it is worth noting that the 2 longest wars in US Army history are the Vietnam War (ie, 17 years, 4 months) and the war in Afghanistan (ie, 17 years at the time of publication); thus, the question of how the length of wars is associated with suicide rates should be a topic for future research. Nevertheless, historical data suggest that combat and increased rates of suicide do not appear to be associated but may be affected by a host of other factors. For example, the population effects of increased racial and gender diversity and inclusion in the US Army over time is an associated factor that deserves to be studied in greater detail. Additionally, service in the US Army does not appear to be inextricably associated with suicide, as rates among active-duty personnel generally decreased until the most recent wars in Iraq and Afghanistan.

According to the *Statistical Report on the Sickness and Mortality in the Army of the United States*,^4^ which covered 1839 to 1855:

During the sixteen years embraced in this report, the army has had but three years’ exemption from field operations and actual hostilities. The war with the Seminole Indians in Florida continued until the summer of 1842; in 1846 war was declared with Mexico, and the forces of the United States were not withdrawn from that country until July, 1848. Since that time the greater portion of the army has been almost constantly employed in long and fatiguing marches, incident to the establishment and occupation of military posts in the newly acquired territories, and to the protection of a greatly extended frontier. Not a year, and, indeed, rarely a month has passed, in which a part of the army has not been engaged in hostilities with some of the numerous and warlike Indians who roam over the great interior plains, or occupy the Pacific slope of the Rocky Mountains.

Soldiering in the 19th century featured the constant threat of war on the frontier, characterized by small-scale, irregular skirmishes similar to some of the hallmarks of modern experiences among service members in the US Army in Afghanistan and Iraq. Additionally, the data available reveal a massive decline in suicide among active-duty personnel in the US Army by the start of World War II (Figure). Following World War II, although suicide rates increased, they failed to reach the prewar rates for the remainder of the 20th century (Figure). This trend ended in 2004, when suicide rates among active-duty personnel in the US Army increased, surpassing pre–World War II rates in 2012 and eclipsing the rate of suicide among civilians.

### Limitations

This report has limitations. While every effort was made to only include active-duty members as categorized by the US Army, that designation presumably included militia service members in the 19th century, and from the 1960s to the 1980s, US Army reporting undoubtedly included individuals who served as reservists as well. Nevertheless, the study focused on tracking suicide rates for active-duty service members in the US Army, however the military chose to define them. These intermittent fluctuations in population should not undermine the study’s integrity. Furthermore, suicide presents a 2-sided and pernicious challenge as a concept of study: completed suicides do not typically document their cause, and tracking the reduction of suicide rates is documenting nonevents. Working with historical data is inherently problematic, and caution is to be urged because, then as now, there is a level of uncertainty in the determination of suicide. However, as Assistant Surgeon General of the US Army J. J. Woodward wrote in 1870 in the *Medical and Surgical History of the War of Rebellion, 1861-1865*, while the medical data “are most imperfect, they embrace so large a proportion of the troops concerned that they cannot fail to serve fairly as a reliable basis for deductions with regard to the health of the whole army.”^6^ Woodward later added that any inconsistencies in data should “not be looked upon as oversights or errors on the part of those to whom preparation of the tables was entrusted. They are the necessary consequence of the fidelity with which the facts, as reported, were consolidated.”^6^ We place faith in the fidelity of historical reporting, as admittedly imperfect as it was. Discussions of what constitutes a fact aside, this study focused on changes and paradigm shifts during long periods more than on attempting to claim with absolute certainty each annual return—something cautioned against even in modern military and medical reporting.

## Conclusions

In this study, the present elevated rates of suicide among active-duty personnel in the US Army served as possible evidence of a pattern that differs from that of the past 200 years. This study, and others like it, could allow for increased testing of causal theories against a longer timeline, considering that, if a model cannot explain the past, it draws into question its prognostic applicability. With the collection of additional historic data sets, researchers may be able to parse out correlation from causation in relation to a host of comparative factors associated with US military suicide.

The consultation of historical data in this study could open to new avenues, dialogues, and collaborations in a more holistic search to better understand suicide. Systematic historical analysis could prepare us to make more informed decisions. The historical perspective of this study provides researchers and policy makers additional opportunities for data and theory analysis as well as increased perspectives. It is a cautious step toward better integration and acceptance of historical frameworks and data from the past 200 years in modern efforts to reduce suicides among active-duty personnel in the US Army.

## Notes

Supplement. **eAppendix 1.** Installation Commanders and Noncombat Casualties **eAppendix 2. **Evolution of Suicide Reporting **eAppendix 3. **Psychology and Combat Exposure **eReferences**
