# Supplementary material for: A Historical Examination of Military Records of US Army Suicide, 1819 to 2017
Source: JAMA Netw Open. 2019 Dec 13;2(12):e1917448. doi: 10.1001/jamanetworkopen.2019.17448 (PMC6991201; doi:10.1001/jamanetworkopen.2019.17448)
Supplement: Supplement. — eAppendix 1. Installation Commanders and Noncombat Casualties eAppendix 2. Evolution of Suicide Reporting eAppendix 3. Psychology and Combat Exposure eReferences [file jamanetwopen-2-e1917448-s001.pdf]

Supplementary Online Content

Smith JA, Doidge M, Hanoa R, Frueh BC. A historical examination of military records of US Army suicide, 1819 to 2017. *JAMA Netw Open*. 2019;2(12):e1917448. doi:10.1001/jamanetworkopen.2019.17448

eAppendix 1. Installation Commanders and Noncombat Casualties

eAppendix 2. Evolution of Suicide Reporting

eAppendix 3. Psychology and Combat Exposure

eReferences

This supplementary material has been provided by the authors to give readers additional information about their work.

© 2019 Smith JA et al. *JAMA Network Open.*

eAppendix 1. Installation Commanders and Noncombat Casualties

Following World War II, the Army became a permanent international fixture. Installation commanders frequently examined noncombat injuries and deaths and adjusted base conditions as necessary to lower them. Frequently, they initiated programs targeting the highest producers of medical and non-combat casualties. They limited alcohol use, reduced speed limits, adjusted training, and followed their senior medical personnel's advice—most often this advice aimed at local environmental pathogens or sexually transmitted diseases, suicide rarely factored into local installation commanders' concerns. In short, throughout the 1950s local installation commanders lent their attention to addressing the primary drivers of noncombat casualties, of which suicide was not a predominant factor.

eAppendix 2. Evolution of Suicide Reporting

Data examination of military suicide has revealed three “waves,” or “evolutions” in suicide reporting history. Broadly, these periods break down into three time periods: 1913 to 1959, 1960 to the mid-1980s, and mid-1980s forward. With the creation of the Department of Defense’s Suicide Event Report in 2007, a potential fourth evolution of suicide research is emerging.

Due in part to their organizational size and their organic medical branch, the U.S. Army was one of the forerunners in public health. The service took great leaps forward in the U.S. Civil War with the creation of the Letterman system and following in the 1870s and 1880s, when it adopted the German General Staff system. Immediately following, the service’s staff functions, to include its medical expertise, professionalized greatly. The Army’s earliest efforts to document and understand the causes of suicide formed the basis of modern military epidemiology. Early Army health specialists took account of age, sex, race, and rank and grade in service. From the earliest data set in 1912 to the late 1950s, Army health specialists primarily documented suicide totals and provided comparative analysis where possible. As will be discussed later in the work, suicide received relatively little attention beyond its documentation during this time period.

Advancements in medicine and public health in the 1950s and 1960s brought greater complexity to the field of medicine and introduced epidemiology to the nascent field of suicide studies. After co-directing the Los Angeles Suicide Prevention Center in 1958, Dr. Edwin Schniedman, a clinical psychologist, founded the American Association of Suicidology in 1968—the association’s founding also introduced, for the first time, the term “suicidologist,” a discipline made of epidemiologists, psychologists, psychiatrists, medical doctors, crises preventionists, social workers, and more.

In the U.S. military, throughout the 1950s and 1960s local and base commanders became increasingly aware of medical threats to the health of the force. On the recommendation of their senior medical officers, the commander set guidance, policies, and even programs to curb chronic medical problems that reduced force strength. In some cases, these bases developed local governance on suicide. The military branches of service turned these policies during the 1970s when, for the first time, the services began nationalizing and centralizing suicide programs.

By the early 1970s, the creation of the All-Volunteer Force and the services’ first “suicide scare” directly led to the creation the first national suicide programs across the branches of service. The creation of the All-Volunteer Force introduced a “market model” to military, which, in addition to establishing recruitment positions within each service, placed an added strain upon them to entice and retain new recruits.

To entice enlistment and maintain service quotas, the U.S. military spent personnel and resources within the recruitment branch of each respective service, as well as offering incentives to potential recruits to insure they join and remain within the service. This placed a greater premium on newly recruited service members. Just two years removed from the creation of the All-Volunteer Force, all branches of service fell victim to a spike in suicides. Of the services, the U.S. Army saw the highest spike at 18 per 100,000 in 1975. As with its sister services, the Army began centralizing and nationalizing previously local suicide prevention programs.

Beginning in the 1960s, the military branches of service conducted research on suicide and began implementing solutions; though each branch of service centralized their guidance and processes, by and large these actions were taken intraservice stove-piped one another. Breakdown of this stove-piping came at the Department of Defense level in 1977, when the over-encumbered Office of the Secretary of Defense separated its administrative and policy-making functions.

While the Office of the Secretary of Defense retained its policy making functions, the newly created Washington Headquarters service took over administration, to include record keeping. Following its creation, the Washington Headquarters Service initiated records management guidelines for all branches of services, which elevated and military suicide data sets to the Department of Defense level.

In the 1980s, equipped with suicide data through the Department of Defense, military epidemiologists examined suicide across the services for the first time. By 1993, the Washington Headquarters Service issued guidance on building a unified suicide report to present before the Joint Chiefs of Staff. The 2008 birth of the Department of Defense’s Suicide Event Report (DODSER) became the modern iteration of joint epidemiological research on suicide for all branches of service at the Department of Defense level. The DODSER was the natural evolution of the Army’s Suicide Event Report (ASER).

eAppendix 3. Psychology and Combat Exposure

Historically, the Army (and Durkheim, in his seminal 1897 book *Suicide: A Study in Sociology*)[1] has not traditionally associated suicide with combat exposure, and there is little evidence to suggest the two are related. For example, high rates in the first part of the 20th century occurred in a peacetime setting. In its analysis of these occurrences, the Army echoed mainstream psychological thought of the time. In its review of the 1920s and 30s, Army medical reports suggested homosexuality as a potential source.[2] Following the publication of Karl Menninger's influential *Man Against Himself* in 1938, the service reflected Menninger's thought when, in the Surgeon General's "Health of the Army" 1945 report, the Army argued "self-destructive impulses" led soldiers to commit suicide by enemy fire.[3] Major wars in Korea, Vietnam, the Gulf, and minor skirmishes in between produced little variance. DODSER data clearly shows a significant portion of Army suicides have no history of deployment.

© 2019 Smith JA et al. *JAMA Network Open.*

eReferences

1. Durkheim E. Suicide: A Study in Sociology. Glencoe, IL: Free Press; 1951.

2. Coffman EM. The Regulars: The American Army 1898-1941. Cambridge, MA: Harvard University Press; 2004.

3. Menninger KA. Man Against Himself. New York, NY: Harcourt Brace; 1938.

© 2019 Smith JA et al. *JAMA Network Open.*
